# Force Fluctuations During Role-Differentiated Bimanual Movements Reflect Cognitive Impairments in Older Adults: A Cohort Sequential Study

**DOI:** 10.1093/gerona/glae137

**Published:** 2024-06-24

**Authors:** Julian Rudisch, Stephanie Fröhlich, Dieter F Kutz, Claudia Voelcker-Rehage

**Affiliations:** Department of Neuromotor Behavior and Exercise, Institute of Sport and Exercise Sciences, University of Münster, Münster, Germany; Department of Neuromotor Behavior and Exercise, Institute of Sport and Exercise Sciences, University of Münster, Münster, Germany; Department of Neuromotor Behavior and Exercise, Institute of Sport and Exercise Sciences, University of Münster, Münster, Germany; Department of Neuromotor Behavior and Exercise, Institute of Sport and Exercise Sciences, University of Münster, Münster, Germany

**Keywords:** Biology of aging, Cognitive decline, Gender differences, Loss of complexity

## Abstract

During role-differentiated bimanual movements (RDBM), an object is typically stabilized with 1 hand and manipulated with the other. RDBM require coupling both hands for coordinated action (achieved through interhemispheric connections), but also inhibition of crosstalk to avoid involuntary movements in the stabilizing hand. We investigated how healthy cognitive aging and mild cognitive impairments (MCI) affect force stabilization during an RDBM in a cohort sequential study design with up to 4 measurement points over 32 months. In total, 132 older adults (>80 years) participated in this study, 77 were cognitively healthy individuals (CHI) and 55 presented with MCI. Participants performed a visuomotor bimanual force-tracking task. They either produced a constant force with both hands (bimanual constant) or a constant force with 1 and an alternating force with the other hand (role-differentiated). We investigated force fluctuations of constant force production using the coefficient of variation (CV), detrended fluctuation analysis (DFA), and sample entropy (SEn). Results showed higher CV and less complex variability structure (higher DFA and lower SEn) during the role-differentiated compared to the bimanual constant task. Furthermore, CHI displayed a more complex variability structure during the bimanual constant, but a less complex structure during the role-differentiated task than MCI. Interestingly, this complexity reduction was more pronounced in CHI than MCI individuals, suggesting different changes in the control mechanisms. Although understanding these changes requires further research, potential causes might be structural deteriorations leading to less efficient (intra- and interhemispheric) networks because of MCI, or an inability to appropriately divert the focus of attention.

Role-differentiated bimanual movement (RDBM) skills are frequently required during tasks of daily living, such as holding a cup in 1 hand and pouring water into it with the other. Such tasks are characterized by a division of labor where 1 hand (typically the nondominant) stabilizes, and the other hand manipulates (1,2). Such RDBM requires that both hands are spatiotemporally coupled, which is achieved through interhemispheric information exchange (crosstalk) via the corpus callosum (3). Previous studies have shown that the integrity of the corpus callosum is important for the coupling between hands (4–6). Additionally, however, interhemispheric neural crosstalk needs to be processed in a task-specific way, involving sensorimotor, but also prefrontal areas of the cortex (7,8). An inefficient inhibition of crosstalk might thus lead to involuntary coupling of the 2 hands (7). The complex intra- and interhemispheric network that is involved in bimanual information processing also makes this function susceptible to changes in the network structure and function, for example, because of aging or cognitive decline, but also due to other factors such as sex (9).

Previous research has shown that healthy older adults perform bimanual coordination tasks with less accuracy and higher performance variability (magnitude of variability) than younger adults (10,11). The analysis also showed that deficits were moderated by the asymmetry and difficulty of the task, with more asymmetric and difficult tasks leading to larger performance deteriorations. Potential causes for these bimanual coordination difficulties are found in the structural changes in the aging brain (12). Functionally, these deteriorations lead to reduced efficiency in (sensorimotor) brain networks. As a result, older adults often show more widespread and diffuse hyper- or hypoactivations which have been discussed to be a signature of either dedifferentiation, compensation, or inefficient processing. Neuropsychologically, compensatory changes are also reflected in the demand for a higher executive control of movement. That is, motor control is processed successively less automated and therewith less in lower areas of the brain and it requires more attentional resources and top-down control (13). Older adults with MCI show even further disconnection of functional brain networks (14) and deficits in executive function (15). This has been shown to lead to differences in interhemispheric inhibition as compared to cognitively healthy peers (16). Consequently, age-related cognitive impairments might further affect interhemispheric information processing and bimanual coordination skills. While a recent scoping review (17) has demonstrated generally impaired (unimanual) upper limb function in older adults with mild cognitive impairment (MCI), there is only scarce evidence on the effects on bimanual coordination. Consequently, it is important to gain further understanding of bimanual coordination deficits in older adults with MCI to understand potential implications on motor behavior and thus understand how MCI differs from cognitively healthy aging with respect to motor skills. This might ultimately also help in finding early-onset markers of age-related cognitive impairments (18).

Besides the magnitude of variability which offers a window into the performance quality of motor control, an assessment of the variability structure (ie, the complexity) of the motor output offers the potential for understanding changes in the control mechanisms of bimanual movements that come with age and disease. In general, healthy individuals demonstrate a certain level of complexity in their motor output. This is considered an indicator of self-organization and the interaction between multiple control loops and redundant degrees of freedom on different levels of the sensorimotor control system (19,20). The complexity of the motor control system is also related to its readiness to adapt to changing constraints as well as perturbations (20,21). Aging and disease are typically associated with a loss of signal complexity and, as such, result in a reduced ability to adapt (21,22). The variability structure of behavioral output is a signature of the system complexity (20–22). In general, 2 domains of measures can be differentiated to assess the variability structure of a signal: information theory-based measures and frequency-based measures (23). Information theory-based measures (eg, sample entropy (24)) assess the temporal patterns or the randomness in a signal. Frequency-based measures typically assess the fractal structure of a signal, that is, the relationship between long- and short-range fluctuations. This can be done, for example, by the use of detrended fluctuation analysis (DFA) (25). Although the measures of fractal scaling and entropy are differentially suited to deal with certain properties of the data, such as nonstationarity (25), they are complementary and typically show a strong negative relationship (20,25). Very rigid systems display high fractal scaling with very few fluctuations on shorter but large fluctuations on longer timescales, and the opposite is true for random systems. Complex physiological systems lie somewhere between these 2 extremes. Previous research has shown that aging leads to lower entropy and higher fractal scaling during a constant force-production task (20), both suggesting an age-related loss-of-signal complexity (21,22).

Additionally, however, the complexity of a behavioral output has been shown to be task dependent. For example, when being asked to modulate their force output in a sine-wave fashion, healthy young adults contrarily display a lower complexity than older adults (20). Similarly, a diversion of attention through a secondary cognitive task has been shown to reduce the randomness in a force output for a force-maintenance task (26). Using measures of performance and coupling, we have previously demonstrated only marginal differences between healthy older adults and those with MCI in a bimanual force-control task with various levels of difficulty and symmetry (27). By the use of DFA, we have shown for younger adults a less complex variability structure of constant force production during a role-differentiated as compared to a bimanual constant task, indicating more rigid control (27). Interestingly, we also found a lesser complexity reduction in the role-differentiated task as compared to the constant task for older as compared to younger adults. To date, the differential effects of aging and MCI on complexity during a bimanual coordination task are unclear, particularly with respect to different requirements of the bimanual task. Based on our previous results, the differentiation between dominant or nondominant hand used for the stabilization task as well as the participants’ sex is also crucial for understanding bimanual task performance (27).

As outlined earlier, role-differentiated tasks require both spatiotemporal coupling and inhibition of crosstalk. In that respect, it is of large interest to focus on the stabilizing hand because a lack of inhibition is expressed in involuntary movements in the stabilizing hand. Such involuntary movements might be related to the presence of MCI (16) and changes in the control due to different task requirements (eg, reduced focus of attention or active inhibition) should also become visible. We, therefore, aimed to investigate both the magnitude of variability as a marker of performance and the structure of variability in force fluctuations during constant force production as a marker of the underlying control mechanisms. We were interested in how they change in the context of a bimanual constant or a role-differentiated task. First, our research objectives were to find out how MCI affects the magnitude and structure of variability, which potentially reflects changes in intra- and interhemispheric information processing (16,28). We hypothesized that (a) the magnitude of variability of MCI participants was higher than that of CHI and that this was even more pronounced in the RDBM than in the bimanual constant task, which reflects general deficits in attention and executive control in MCI and this is exacerbated with increasing task difficulty (15); (b) complexity was overall lower in MCI as compared to CHI, being a signature of a loss of physiological complexity (21); (c) complexity (as measured through fractal scaling and entropy) was reduced in the role-differentiated as compared to the bimanual constant task, being a signature of interhemispheric crosstalk (7); and (d) this complexity reduction is larger in MCI as compared to CHI, being a signature of increased interhemispheric crosstalk or lack of inhibition in the MCI group (16). Second, we wanted to investigate whether these effects become more pronounced over time due to the further progression of aging and potentially related cognitive decline. We assumed to find a progression of variability measures across an 8–32 months time interval.

Our secondary objectives were to investigate differences between dominant and nondominant hands, as well as potential sex effects. In line with our previous research, we hypothesized that the nondominant hand shows better performance with a lower magnitude of variability and higher complexity in the role-differentiated task and that females show higher fractal scaling than male participants (27).

## Method

### Study Design and Setting

Data collection was part of the SENDA (sensor-based systems for early detection of dementia) study (18) which was designed as a sequential cohort study. Participants were tested between 1 and 4 times over the duration of 3 years with intervals of 8 months between visits. Data collection started in January 2018 and finished in March 2020. Testing took place in the lab premises of Chemnitz University of Technology, Germany.

### Participants

**Figure 1.**
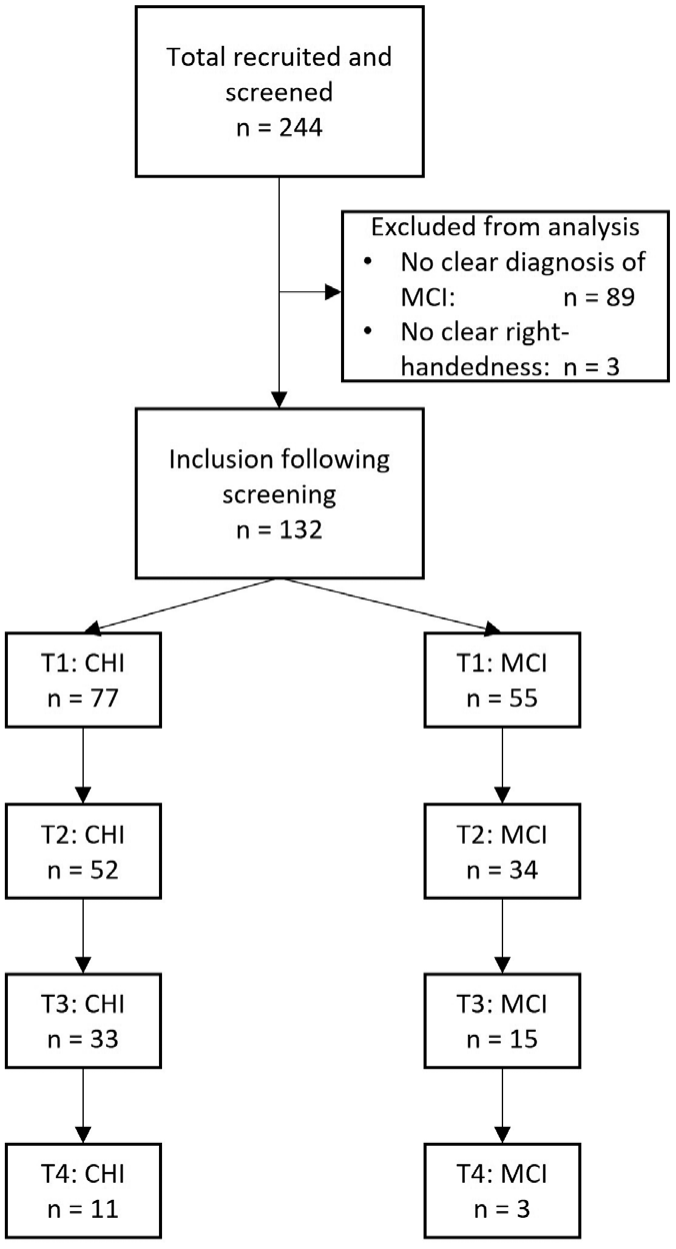
Flowchart of participants included in the analysis prior to and following screening for eligibility and for the different timepoints (T1—T4) in the group of cognitively healthy individuals (CHI) and those with mild cognitive impairments (MCI).

We recruited community-dwelling older adults (aged ≥80 years) through local advertisements in newspapers in Chemnitz (Germany). Participants were included if they were community dwelling, able to independently travel to the testing premises, and able to understand instructions. Individuals were excluded from the study if they had been diagnosed with psychological or neurocognitive disorders. Additionally, in this study, we only included those participants who were determined right handed (scores >40) by the Edinburgh Handedness Inventory (29) and excluded ambidextrous individuals and left handers. The study was approved by the Research Ethics Committee at the Faculty of Behavioral and Social Sciences at Chemnitz University of Technology (V-232-17-KM-SENDA-07112017). See Figure 1 for a flowchart of the recruitment process and dropout at different time points.

### Experimental Methods and Procedures

#### Apparatus

We used 2 compression load cells (29.5 mm diameter, 8 mm depth, 0–22 N measurement range) inside a 3D-printed structure to measure pinch-grip forces. Signals were amplified and subsequently converted to digital format using a NI-DAQ USB 6002 (National Instruments, Austin, TX) and sampled at a frequency of 120 Hz. Participants sat in front of a screen (60 cm distance, 23.8-inch monitor, hardware resolution 1,920 × 1,080 pixels, 60 Hz updating frequency), holding the 2 force transducers using a pinch grip, 1 in each hand. We used a customized LabView program for experimental procedures. The program allowed participants to observe real-time feedback of their measured forces on the screen in the form of 2 yellow dots moving up or down when applying or releasing force. Target forces were displayed in the form of 2 square shapes (12.5mm width and height). A more detailed description and visualization of the setup were published previously (18,27).

#### Procedures

##### Screening

The German version of the Montreal Cognitive Assessment (MoCA (30)) was used to measure global cognitive functioning. In addition, the German version of the Consortium to Establish a Registry for Alzheimer’s Disease Neuropsychological Test Battery (CERAD-NP (31)) was used to examine the cognitive domains memory, language, executive functions, and visuo-construction. Lastly, the years of education were recorded to apply the correction point to the MoCA for all individuals with 12 or less years of education. MCI was determined according to the performance in the MoCA (1 sum score) and 9 CERAD-NP test scores: fluency (number of animals named in 1 minute), Boston naming test (number of objects correctly identified), phonematic fluency (number of words named with letter “S” in 1 minute), constructional praxis (number of correctly copied characteristics), word list learning (number of words correctly remembered in the third trial), word list recall (savings score), word list recognition (discriminability score), constructional praxis recall (savings score), and trail-making test (quotient B/A). We followed a 2-step procedure that is recommended for the diagnosis of MCI in the general population, which states that, first, a screening should be used, and, in case of abnormal findings, in-depth cognitive testing should follow (32). This strategy was applied successfully in prior analyses of the same sample (33). A MoCA score below 26 points (30) and at least 1 CERAD-NP performance below 1.5 standard deviations (*SD*s) of the normative mean (taking into consideration age, sex, and education level) resulted in the classification of MCI. All other participants were classified as cognitively healthy individuals (CHI).

##### Bimanual force-control experiment

Initially, participants performed a test of maximum voluntary contraction (MVC) with their dominant hand by pressing a force transducer as strong as possible for a duration of 5 seconds. The procedure was repeated 3 times and the peak measured value was used as MVC. Subsequently, the participants completed a series of conditions including bimanual constant and role-differentiated tasks, but also inphase and antiphase coordination modes (27). For this study, we only analyzed the bimanual constant and role-differentiated modes.

For the bimanual constant condition, the target force for both hands was set to a constant level of 12% of the individual MVC. For the role-differentiated condition, the target force of 1 hand was set to a constant level of 12%; that of the other hand varied following a sine-wave function with a frequency of 0.2 Hz and a range of 5%–12% of MVC. Participants had to match target forces (indicated by a square rectangle on the screen) by keeping their force levels (indicated by a small dot) inside the targets. Although previous studies with older adults (up to 65 years of age) have selected higher target forces of, for example, 5%–25% (13), we have selected a lower target force to avoid fatigue because we included highly aged participants and the study design consisted of a large number of trials in different conditions (18).

**Figure 2.**
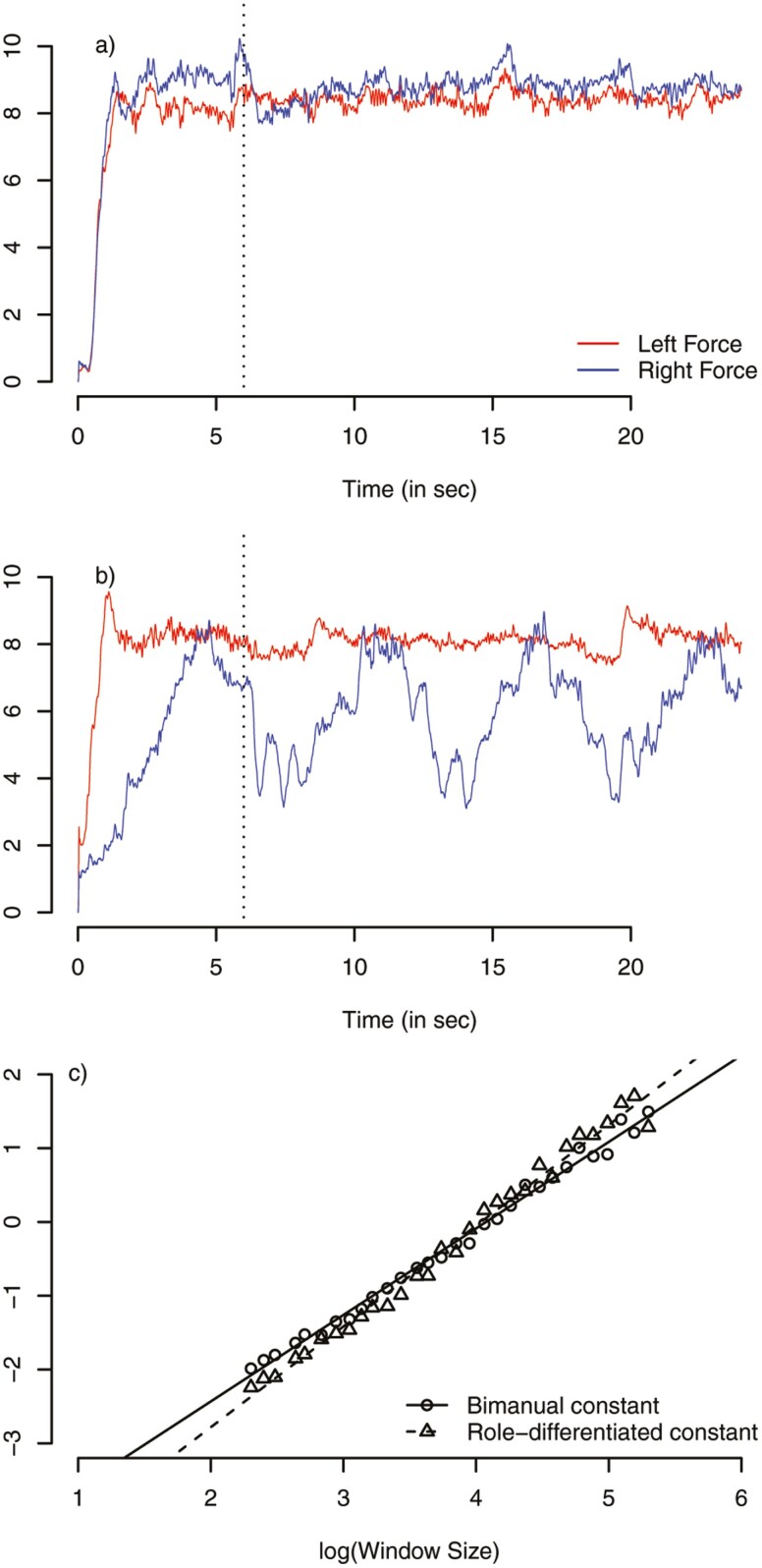
Exemplary raw data from a single participant in the (A) bimanual constant and (B) role-differentiated condition. The dotted vertical lines in (A) and (B) display the exclusion of the initial 6 seconds of each trial. (C) Log-linear relationship between the root-mean-square error (RMSE) and increasing window sizes for the force data of the left hand in the 2 conditions bimanual constant and role-differentiated as result of the detrended fluctuation analysis (DFA).

Based on previous studies investigating pinch-grip force control in older adults (34,35), the trial duration was 20 seconds. Participants completed 2 trials in the bimanual constant and 8 trials in each of the role-differentiated conditions where the dominant or nondominant hand produced the stabilizing force. The initial 6 seconds (ramp phase) were excluded from the analysis, however. Figure 2 exemplarily shows measured signals for the 2 hands in the 2 conditions of bimanual constant (Figure 2A) and role-differentiated (Figure 2B).

### Data Analysis

#### Preprocessing and outcomes

Data analysis was performed in R version 4.2.2 (36). Nonlinear measures of fractal scaling (DFA) and entropy were computed with the package “nonlinearTseries” (37).

##### Magnitude of variability: coefficient of variation

To assess the magnitude of variability, coefficient of variation (CV) of each time series (*x*) was computed as the quotient of the *SD* divided by the mean of the time series, multiplied by 100:


$$\begin{array}{l} CV_{x}=\;\frac{SD_{x}}{\bar{x}}\times 100 \end{array}$$

##### Structure of variability

We used 2 measures to assess the variability structure (complexity) of a time series. Fractal scaling by the use of detrended fluctuation analysis (DFA-α) reflects the relationship between high- and low-frequency fluctuations. Sample entropy (SEn) reflects the (un)predictability of a time series. With a decrease in complexity (ie, from white noise to Brownian noise), DFA-α increases and SEn decreases.

##### Fractal scaling: detrended fluctuation analysis

For DFA, we used the algorithm proposed by Peng et al. (25). The time series is, therefore, integrated and then separated into nonoverlapping segments of equal length (*m*). A linear model is fitted to each segment and the root-mean-square deviation from the model is calculated (average over all segments). The same is done for increasing window sizes *m*. The log-linear relationship (slope coefficient α) between the average root-mean-square deviation F(m) and the window size m is a measure of the fractal scaling:


$$\begin{array}{l} F\left(m\right)=\;m^{\alpha } \end{array}$$

Uncorrelated random time series typically demonstrate smaller scaling coefficients (eg, α ≈ 0.5 for white noise). On the other hand, higher scaling coefficients demonstrate self-correlations in the signal (α ≈ 1.5 for Brownian noise). Complex physiological signals lie somewhere between these 2 extremes, showing some amount of self-correlation but also some random fluctuations (25).

To compute DFA in this study, we used 30 different exponentially increasing window sizes *m* between lengths 10 and 200. Figure 2C depicts exemplary DFA results for 1 participant in the 2 different conditions. In the role-differentiated condition, force fluctuations in the stabilizing hand are smaller (higher noise) on short timescales but increase more quickly with larger fluctuations on longer timescales (solid line in Figure 2C) as compared to the bimanual constant condition (dashed line).

##### Randomness: sample entropy

Entropy measures quantify the predictability or randomness in a time series. That is, based on what we know about the current state of a time series, can we predict the future state? Signals that are more predictable have a low entropy whereas the opposite is true for random signals (eg, white noise). For the computation of SEn, it first needs to be quantified how many segments of length *m* are similar to other segments with the same length in the time series. Similarity is established by computing the Euclidean distance between segments verifying whether the distance is above or below a threshold *r*. The conditional probability for the occurrence of similar segments is then computed for a segment length m ($C_{m}$) and a segment length *m* + 1 ($C_{m+1}$). The SEn is then computed as a natural logarithm of the likelihood ratio:


$$\begin{array}{l} SEn=\;ln\left(\frac{C_{m}}{C_{m+1}}\right) \end{array}$$

The rationale behind this procedure is that, if signals are perfectly repeatable, the likelihood of finding similar segments with length *m* is similar as compared to *m* + 1. On the other hand, for random signals, the likelihood to find similar elements drops substantially if the segment length is increased. Therefore, random signals have a much higher likelihood ratio (SEn > 2 for white noise) as compared to deterministic time series (SEn < 0.5 for Brownian noise) (38). In this study, we scaled signals, so they have the same mean and *SD*. We then used a segment length of *m* = 2 and a radius of *r* = 0.25. To ensure the robustness of this parameter choice (24), we have additionally computed SEn for neighboring parameters (*m* = 3 and 4; *r* = 0.2 and 0.3) and added the results for the statistical analysis to Supplementary Tables 4 and 5.

### Statistical Analysis

We used the “nlme” package (39) to compute linear mixed effects models. We investigated the impact of condition (bimanual constant vs role-differentiated), group (CHI vs MCI), hand (left vs right), sex (male vs female), and timepoint (t1–t4) on the different outcomes. Participant ID was used as random effect to account for the repeated-measures design. We stepwise included the main effects and all 2-way interactions. Additionally, we added 3-way interactions between condition, group, and timepoint, as well as condition, group, hand, and sex. Model fit was analyzed from the log-likelihood and Akaike Information Criterion (40). Likelihood ratios were computed as a measure of effect sizes and statistically tested according to Field, Miles, and Field (41) and are reported in Supplementary Tables 1–3. We further selected the model with the best fit (lowest AIC) to inspect individual model contrasts and reported betas with standard errors, degrees of freedom, *t*- and *p* values, as well as effect sizes *r*.

## Results

### Participants

We included 132 participants in this study, 77 of which were classified as CHI and 55 as MCI at the first assessment. Due to the cohort sequential study design, the number of participants differed significantly at every timepoint (see Figure 1 for a flowchart). Group characteristics at the different timepoints are shown in Table 1. There were small but significant age differences between the 2 groups at T1 (*t*(99) = −1.99, *p* = .049). The proportion of female participants was slightly larger in the group of CHI, but differences were not statistically significant. No significant differences between groups were found for MVC. CHI had significantly larger MoCA scores at the first measurement point (*t*(89) = 18.40, *p* < .001) and scores remained stable over time. As opposed to that, MoCA scores in the MCI group improved over time (3 points between the first and last measurement points).

**Table 1.** Participant Group Characteristics and Outcomes Across Different Timepoints

	T1		T2		T3		T4	
Group	CHI	MCI	CHI	MCI	CHI	MCI	CHI	MCI
*N*	77	55	52	34	33	15	11	3
Age at measurement (mean [*SD*])	82.00 [2.28]	82.93^a^ [2.80]	82.55 [2.11]	83.69 [2.90]	83.22 [2.49]	83.99 [3.38]	83.34 [1.35]	82.55 [1.34]
Female (*n* [%])	42 [55.3%]	25 [46.3%]	31 [59.6%]	17 [50.0%]	18 [54.5%]	4 [26.7%]	6 [54.5%]	1 [33.3%]
MVC in *N* (mean [*SD*])	23.9 [7.9]	22.1 [8.2]	23.0 [6.8]	25.3 [10.0]	25.3 [8.0]	25.3 [9.5]	24.7 [8.5]	22.0 [9.9]
MoCA Scores (mean [*SD*])	27.7 [1.2]	22.6^b^ [1.8]	26.8 [2.3]	23.6^b^ [2.3]	27.1 [2.1]	24.0^b^ [1.6]	27.8 [2.1]	25.6 [2.1]
CV (mean [*SD*])	6.65^a^ [4.76]	8.32 [6.46]	6.67 [5.81]	6.99 [4.32]	6.46 [4.32]	6.66 [4.83]	5.34 [2.47]	7.28 [4.75]
DFA-α (mean [*SD*])	1.384 [0.156]	1.395 [0.139]	1.384 [0.150]	1.394 [0.125]	1.376 [0.140]	1.362 [0.139]	1.352 [0.159]	1.338 [0.142]
SEn (mean [*SD*])	0.345 [0.155]	0.334 [0.165]	0.364 [0.176]	0.334 [0.180]	0.352 [0.150]	0.368 [0.154]	0.380 [0.123]	0.380 [0.176]

*Notes*: CHI = cognitively healthy older adult; CV = coefficient of variation; DFA-α = scaling exponent of detrended fluctuation analysis; MCI = individuals with mild cognitive impairments; MoCA = Montreal Cognitive Assessment Score; SEn = Sample Entropy; T1–T4 = Measurement points 1–4. ^a^ = *p* value < .05; ^b^ = *p* value < .001 (between group differences were tested using Welch’s 2-sample *t*-test for metric variables and χ^2^ statistics for count data).

### Outcomes

**Figure 3.**
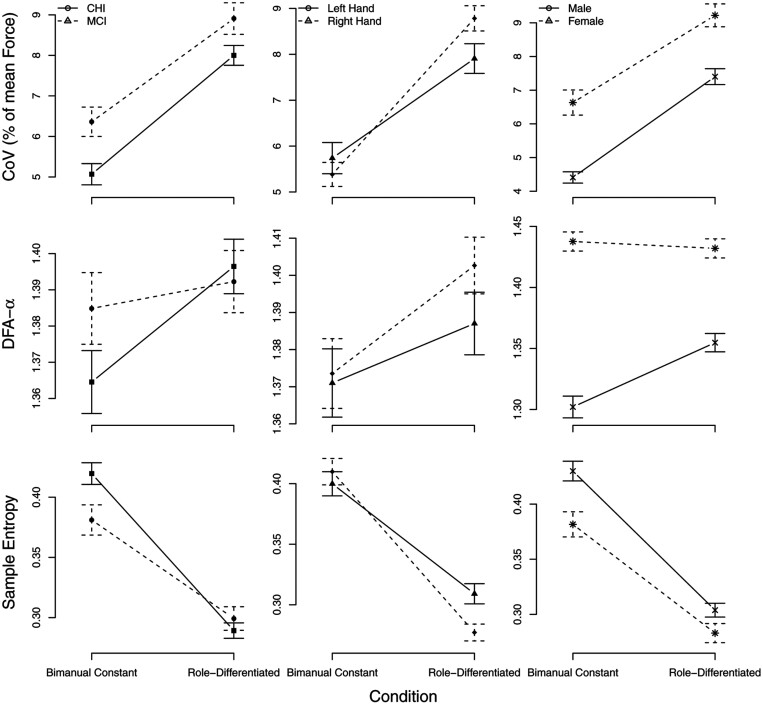
Interaction effects with the factor condition for the different outcomes coefficient of variation (first row), detrended fluctuation analysis (second row), and sample entropy (third row). Graphics show mean values and standard errors (*SE*s) for each condition, aggregated for groups (first column); the left and the right hand (second column), and the sex (third column). Error bars indicate 1 *SE*).

Model effects for the different outcomes are presented in Table 2. Interaction effects are displayed in Figure 3.

**Table 2.** Linear Mixed Model Results for the Outcomes Coefficient of Variation, Detrended Fluctuation Analysis, and Sample Entropy

Coefficient of Variation						
	Beta	Std. Error	*df*	*t*	*p* Value	*r*
Intercept	4.33	0.67	553	6.44	**<.001**	**0.26**
Condition (RD–BC)	2.40	0.47	129	5.13	**<.001**	**0.41**
Group (MCI–CHI)	1.76	0.75	128	2.33	**.021**	**0.2**
Hand (right–left)	−0.36	0.24	553	−1.47	.142	0.06
Sex (female–male)	2.20	0.66	128	3.34	**.001**	**0.28**
Timepoint	−0.10	0.16	293	−0.65	.515	0.04
Condition (RD–BC) × Group (MCI–CHI)	−0.42	0.69	129	−0.61	.54	0.05
Condition (RD–BC) × Hand (right–left)	1.24	0.34	553	3.62	**<.001**	**0.15**
Detrended fluctuation analysis						
	Beta	Std. Error	*df*	*t*	*p* Value	*r*
Intercept	1.302	0.020	552	66.1	**<.001**	**0.94**
Condition (RD–BC)	0.043	0.017	128	2.61	**.010**	**0.22**
Group (MCI–CHI)	0.018	0.021	128	0.83	.408	0.07
Hand (right–left)	−0.006	0.007	552	−0.89	.376	0.04
Sex (female–male)	0.141	0.021	128	6.81	**<.001**	**0.52**
Timepoint	−0.003	0.005	292	−0.58	.560	0.03
Condition (RD–BC) × Group (MCI–CHI)	−0.028	0.014	128	−2.07	**.040**	**0.18**
Condition (RD–BC) × Hand (right–left)	0.013	0.009	552	1.47	.141	0.06
Condition (RD–BC) × Sex (female–male)	−0.054	0.013	128	−4.04	**<.001**	**0.34**
Condition (RD) × Timepoint	0.006	0.006	292	0.96	.335	0.06
Group (MCI–CHI) × Hand (right–left)	0.023	0.009	552	2.56	**.011**	**0.11**
Sample entropy						
	Beta	Std. Error	*df*	*t*	*p* Value	*r*
Intercept	0.419	0.020	553	20.73	**<.001**	**0.66**
Condition (RD–BC)	−0.107	0.014	129	−7.50	**<.001**	**0.55**
Group (MCI–CHI)	−0.043	0.022	128	−1.91	**.058**	**0.17**
Hand (right–left)	0.010	0.007	553	1.45	.147	0.06
Sex (female–male)	−0.027	0.019	128	−1.39	.167	0.12
Timepoint	0.003	0.005	293	0.50	.620	0.03
Condition (RD–BC) × Group (MCI–CHI)	0.048	0.021	129	2.22	**.028**	**0.19**
Condition (RD–BC) × Hand (right–left)	−0.042	0.010	553	−4.28	**<.001**	**0.18**

*Notes*: BC = bimanual constant; CHI = cognitively healthy older adult; *df* = degrees of freedom; MCI = individuals with mild cognitive impairments; RD = role-differentiated; *r* = effect size (Pearson’s *r*); *t* = *t*-statistic. Note that for detrended fluctuation analysis, the model with the best fit contains additional interaction effects as compared to coefficient of variation and sample entropy.

#### Coefficient of variation

For CV, inspection of the main effects showed: (a) a significantly higher CV in the role-differentiated condition compared to bimanual constant (*t*(129) = 5.13, *p* < .001, *r* = 0.41); (b) overall larger CV in MCI compared to CHI (*t*(128) = 2.33, *p* = .021, *r* = 0.20); and (c) overall larger CV in females as compared to males (*t*(128) = 3.34, *p* = .001, *r* = 0.28). Interaction effects (Figure 3) showed no CV differences between the left and right hands in the bimanual constant condition, but a higher increase in CV in the right than in the left hand when performing the role-differentiated condition (*t*(553) = 3.62, *p* < .001, *r* = 0.15).

#### Detrended fluctuation analysis

For DFA-α, inspection of the main effects showed (a) a lower (less complex) DFA-α in the bimanual constant than the role-differentiated condition (*t*(128) = 2.61, *p* = .010, *r* = 0.22); and (b) larger DFA-α in females than males (*t*(128) = 6.81, *p* < .001, *r* = 0.52). Interaction effects (cf, Figure 3) showed (a) a lower DFA-α for MCI than CHI in the bimanual constant condition, but a higher DFA-α for MCI in the role-differentiated condition (*t*(128) = -2.07, *p* = .040, *r* = 0.18); (b) an increase of DFA-α for male participants from bimanual constant to role-differentiated, but an overall high DFA-α for female participants (*t*(128) = -4.04, *p* < .001, *r* = 0.34); and iii) similar values for DFA-α for both hands in CHI but a larger DFA-α in MCI in the right hand (*t*(552) = 2.56, *p* = .011, *r* = 0.11).

#### Sample entropy

For SEn, inspection of the main effects showed (a) a lower entropy (higher signal predictability) in bimanual constant than role-differentiated condition (*t*(129) = −7.50, *p* < .001, *r* = 0.55); (b) lower entropy in the MCI group than CHI (*t*(128) = −1.91, *p* = .058, *r* = 0.17). Interaction effects (Figure 3) showed (a) higher entropy for CHI as compared to MCI in the bimanual constant condition and a vice versa pattern in the RD condition (*t*(129) = 2.22, *p* = .028, *r* = 0.19); and (b) higher entropy for the right hand as compared to the left in the bimanual constant condition and vice versa in the role-differentiated condition (*t*(553) = −4.28, *p* < .001, *r* = 0.19).

## Discussion

In this study, we investigated the magnitude and structure of variability during a role-differentiated bimanual force-control task in 80+-year-old older adults with and without MCI in a time interval of up to 32 months. We were interested in whether the magnitude and structure of variability are affected by cognitive decline and whether any further deteriorations are observable over time in a sequential cohort study design. Our results showed that older adults with MCI (compared to CHI) generally showed a higher magnitude of variability, as indicated by the CV, in both the bimanual constant and role-differentiated condition. An investigation of the structure of variability through DFA and sample entropy, however, showed a more differentiated picture. That is, (a) CHI showed a more complex variability structure with higher entropy and lower DFA-α than MCI in the bimanual constant condition; (b) whereas both groups reduced complexity in the role-differentiated condition as compared to the bimanual constant, (c) CHI showed significantly larger reductions, resulting in a less complex variability structure for CHI than MCI in the role-differentiated condition. Contrary to our expectations, we did not find any changes in the magnitude or structure of variability over time in either of the groups that could reflect a progression of age- or disease-related decline.

### Lack of Inhibition or an Internal Focus of Attention Might Lead to a Decreased Complexity of Constant Force Production During a Role-Differentiated Task

A more random (complex) variability structure in the force signal is associated with larger degrees of freedom and self-organized control (20). Consequently, the opposite signifies a more rigid, top-down control. Our results showed lower entropy for constant force production in the role-differentiated as compared to the bimanual constant task, reflecting an increased regularity in the signal. In line with this, we found an increased fractal scaling in the role-differentiated as compared to bimanual constant tasks, suggesting a shift toward slower control mechanisms and less self-organization. We have previously also shown such a task-dependent shift in the fractal scaling for young adults (27).

Previous research has shown that the complex variability structure is also highly dependent on the task demands. For example, when the task is to stabilize a certain output around a fixed point (force stabilization), the complexity is typically high. As opposed to that, if the task demands are of oscillatory nature (eg, producing a sine-wave pattern), the complexity is reduced (20,27). Lower complexity in constant force production in the role-differentiated as compared to the bimanual constant condition suggests freezing of degrees of freedom in the role-differentiated condition. One potential reason for this might be a shift in the focus of attention. The constrained action hypothesis states that an external focus of attention allows the system to self-organize more naturally, utilizing more degrees of freedom (42) by controlling the external effect of a task in a feedback fashion. This is, as opposed to an internal focus of attention where the specifics of the movement plan are controlled in a more top-down fashion, requiring constraining degrees of freedom. The bimanual constant task, in fact, might allow for a more externalized focus of attention because the symmetric and simple task makes it easy to integrate visual feedback. On the other hand, the role-differentiated task might require an allocation of attentional resources toward the sine-wave task, therefore, necessitating control of the constant force production in a feedforward manner (which might be an efficient solution for this task). Previous research has, in fact, shown that a reduction of attentional resources through a secondary cognitive task reduces the complexity of processes in an unimanual visuomotor force control task (43,44), which is likewise argued to result in an internalized focus of attention.

Another explanation might be that an involuntary coupling of the 2 hands due to reduced interhemispheric inhibition might lead to a mirroring of the sine-wave force production in the stabilizing hand (mirror movements). Although mirror movements are predominantly observable across various patient populations (45,46), they can also be found in healthy persons and are experienced particularly by children or highly aged older adults (47). The presence of mirror movements in nonclinical populations suggests inefficient processing of crosstalk (45). However, younger adults also show less complexity (27) but are not prone to mirror movements.

Interestingly, our results also demonstrated that the directionality of the role-differentiated task has a strong impact on both, the magnitude and structure of variability of force stabilization. Although no differences were found between the dominant and nondominant hand during the bimanual constant task, we found an increased magnitude of variability and decreased complexity in the dominant hand during the role-differentiated task. In sum, these results indicate better control over the nondominant than the dominant hand during role-differentiated tasks. Although this is somewhat counterintuitive, previous research also indicates that the assignment of the stabilizing role to the nondominant hand is, in fact, preferred for role-differentiated movement tasks (1,2). Consequently, a larger reduction of complexity in the nonpreferred condition indicates 2 potential reasons (following the stream of argumentation earlier): (a) poorer inhibition during the nonpreferred task condition, leading to even increased mirror movements; or (b) the nonpreferred condition requires even higher focus of attention on the manipulation (sine-wave task) and diversion of attention with more rigid top-down control for the constant force production. In fact, there is evidence that mirror movements are increased in the dominant hand when the nondominant hand is performing a certain task (27,48). This asymmetry has also been shown in neurophysiological studies (7,49) and suggests that the left hemisphere is more excitable or functional inhibition is reduced.

### Older Adults With MCI Show Higher Complexity in the Role-Differentiated Task and Lower Complexity in the Bimanual Constant Task as Compared to CHI

Our study also showed that cognitive decline affects both the structure and the magnitude of variability of constant force production. That is, those with MCI show generally higher CV, reflecting reduced performance accuracy and less stable performance. Interestingly, for the structure of variability, we found an interaction between groups and conditions with the CHI group displaying a more complex signal (higher entropy, lower DFA-α) in the bimanual constant but less complexity in the role-differentiated condition as compared to MCI. A reduced complexity in the bimanual constant task is in line with the hypothesis that age and disease lead to a reduction of physiological complexity and consequently a reduced ability of the (sensorimotor) system to adapt (20–22,50). In the case of this task, this loss of complexity might be traced back to changes on various systems levels, including motor (eg, a reduction of neuromuscular degrees of freedom), sensory (eg, a reduced number of receptors), or cognitive changes (eg, a lack of attentional resources). Although it might be speculated that these group differences are driven by a lower target force, we did not find any group differences in the MVC and therefore the average relative target force (cf, Table 1).

Interestingly, however, individuals with MCI displayed a more complex variability structure during the role-differentiated task. Although this result is counterintuitive as it typically is associated with a healthier system, we have previously shown that also younger adults show a much stronger reduction of complexity in a role-differentiated task as compared to older adults (27) and interpreted it as more rigid control. Therefore, a lower complexity in the role-differentiated task might reflect the ability to adapt to changing task demands, rather than a loss of complexity in sensorimotor control systems (20). As pointed out earlier, a reduction of complexity might be due to a shifted focus of attention toward the manipulating hand. Following this line of argumentation, the group differences could reflect that individuals with MCI show a reduced ability for top-down control and to inhibit crosstalk or that the CHI group allocate more attentional resources to the manipulation.

MCI might affect bimanual information processing in several ways. For example, demyelination of the corpus callosum might lead to a functional disconnection of the 2 hemispheres (16,28). This would result in a decoupling of the 2 hemispheres (6), which might bring about an advantage for a lower demand to inhibit crosstalk (while at the same time having negative effects on the spatiotemporal coupling of the hands (4)). Contrarily, however, inhibition of crosstalk might be impaired due to deteriorations of sensorimotor networks and executive dysfunctions (51). In fact, we have previously shown no differences in the coupling between hands during role-differentiated tasks in older adults with and without MCI (27), and to our knowledge, no study exists that provides evidence for extensive mirror movements in older adults with cognitive dysfunctions. Another possible explanation for the lower complexity in CHI might be that cognitively healthy adults allocate their attentional focus on the sine-wave task and are able to fix their constant force production level to a certain degree without constant visuomotor feedback (top-down control). However, in a previous study, we have also shown no performance differences in the sine-wave force production (27). Overall, it is, however, evident that CHI are more similar to young and healthy adults (27) by showing stronger reductions of complexity in constant force production during a role-differentiated as compared to a bimanual constant task.

### Complex Variability Structure Does Not Show Significant Changes Over the Course of 3 Years

Despite our hypothesis, we did not find any evidence for further changes in the variability structure that might reflect a loss of complexity of the sensorimotor control system as a consequence of age and disease (22). One explanation might be that effects of a complexity loss are camouflaged by opposing effects through motor learning of the task. That is, participants visited our laboratory a maximum of 4 times over the period of 3 years.

Indeed, although it is generally accepted that aging is accompanied by a loss of complexity (21,22), there is a lack of evidence from longitudinal studies on age-related changes in the variability structure of motor control processes. Cross-sectional studies comparing young and older adults have shown, however, that aging leads to a reduced complexity with higher fractal scaling (20,27) and lower entropy (20,52). However, it has also been previously shown that older adults can retain complexity in finger force production due to practice when they were exposed to increased practice, for example, as a consequence of professional labor (52). It is, therefore, likely that motor practice may be able to restore and increase complexity even in older ages. In fact, it has been shown that practice over 5 consecutive sessions leads to an increase in the entropy during a constant fore production task and a decrease in complexity in a sine-wave force production task in both young and old adults (53).

One additional factor that might be considered, however, is a selection bias. Our study demonstrated high dropouts over time. It is likely that exactly those participants dropped out of the study who were confronted with sudden changes to their health status and were thus not able to participate in the study anymore. Therefore, the longitudinal cohort consisted of rather robust individuals who might not display any strong health deteriorations.

### Females Show High Fractal Scaling Across Conditions

Lastly, the findings require some discussion of the effects of sex upon fractal scaling of signals. Although sex did not show an effect on sample entropy, it did show a strong effect on the CV as well as the DFA-α, with female participants showing a larger magnitude and increased fractal scaling, particularly in the bimanual constant condition. One potential factor driving these sex differences might be the significantly lower MVC found for female participants and the fact that we used target forces relative to that. A previous study by Svendsen and Madeleine (54) made similar observations, showing higher magnitude and lower complexity for female participants in an isometric elbow flexion task with target forces relative to the MVC. Physiologically, these results might be rooted in neuromuscular and musculoskeletal differences between female and male participants. It has been shown for the first dorsal interosseus that males have larger proportions of fast-twitching (type II) muscle fibers (55). Female muscles, on the other hand, display higher firing rates as compared to males already at submaximal contractions (55). This could be an indicator of an earlier recruitment of a larger proportion of muscle fibers in females, whereas males sustain a higher reserve of muscle fibers, that is, more degrees of freedom that might be recruited. This interpretation is, however, highly speculative and necessitates further research on the biological factors causing these sex effects. This is especially important because sex differences are only observable in the DFA-α, but not in the sample entropy, even though both measures typically show a strong negative relationship. Future research should focus in elucidating why the sex effects are picked up differentially by the 2 measures. Additional sex-related factors such as an increased joint laxity and a reduced stiffness in the muscle-tendon properties (56) might result in less direct feedback processing, which might, in turn, be reflected in the fractal scaling of the force output.

Interestingly, and contrary to the general pattern, female participants did not show an increased fractal scaling in the role-differentiated as compared to the bimanual constant condition. As pointed out earlier, female participants already display high fractal scaling in the bimanual constant condition. Therefore, the role-differentiated condition might not result in any additional reduction of complexity (ceiling effect). However, previous research has indeed also shown sex-related differences in bimanual coordination attributed to differences in the central nervous system, with female participants showing greater interhemispheric functional connectivity (9). Although these sex-related differences in interhemispheric information processing might be related to our strong sex effects, they are likewise speculative and warrant further research relating neuroimaging with behavioral measures.

### Limitations

Although our study is of importance by demonstrating that MCI has a strong effect on the complexity of force production during different bimanual force-control tasks, therefore, suggesting that cognitive disorders affect bimanual information processing, several factors limit the interpretation of our results. No decisive conclusion can be made on the underlying mechanisms that lead to the more pronounced complexity reduction in healthy older adults as compared to those with MCI. While two potential mechanisms are discussed, that is, (a) an absence of interhemispheric crosstalk in individuals with MCI and (b) a more externalized attentional focus on the constant force production in the role-differentiated task for MCI, further research for example involving neuroimaging methods and other methods to divert attention, such as dual-tasking paradigms are necessary to gain more insights. Despite the original aim of investigating longitudinal changes in the complexity of a cohort study design, we did not find any changes over time. The study required participants to be able to independently travel to and attend the measurement sessions. Therefore, it is possible that a selection bias occurred which filtered out those individuals that dropped out due to stark deteriorations in their physical state. Future studies that investigate longitudinal changes might, therefore, consider testing not in a lab but in the living environment of the participants, to reduce the selection bias. One aspect that might have biased the results was the comparably low sample size at timepoints T3 and T4, owed to the cohort sequential study design. Analysis of a reduced data set excluding these timepoints did not significantly affect the results, however (see Supplementary Tables 6–8). However, the low sample size at these timepoints also limits the power of the statistical analysis to detect time effects. One further limitation that might reduce the comparability of our results with other studies that calculate sample entropy is the length of individual trials. Although previous studies have used even shorter trials to compute entropy from pinch-grip force control data in older adults (20,43), it has been suggested to use trial lengths of at least 30 seconds (57) to capture all biological phenomena hidden in the variability structure of force-control data. However, the trade-off for increasing the trial duration, particularly for older adults, might be fatigue which would be another factor severely affecting the complexity of force output (44).

## Conclusion

Our study has demonstrated that age-related cognitive impairments affect the magnitude and structure of variability of constant force production during bimanual constant and role-differentiated movement tasks. Contrary to our expectations, we did not find any changes in both the magnitude and structure of variability over time (over the course of a maximum of 3 years), which might potentially reflect further progression of disease. Our results showed, however, that the use of different measures of the magnitude and structure of variability can reveal different aspects of task- and disease-related effects upon bimanual control. As expected, we did find a higher magnitude of variability in more difficult tasks and in participants with MCI. On the other hand, both the DFA and SEn showed a higher complexity (which is typically associated with a healthier system) in CHI in the bimanual constant condition than MCI and provided a more differentiated picture. Both measures were able to depict that CHI also displayed a more pronounced complexity reduction, indicating a more rigid control with fewer degrees of freedom. Future studies should investigate whether this is a top-down processes related to the cognitive functions of an individual and/or to a change of attentional focus (eg, through instructions or a secondary cognitive task). Additionally, neurophysiological measures of task-related information processing (eg, EEG or fMRI) might reveal whether a reduction of complexity can be related either to an increased interhemispheric crosstalk or active inhibition thereof.

## Supplementary Material

glae137_suppl_Supplementary_Materials

glae137_suppl_Supplementary_Data
