# Supplementary material for: Force Fluctuations During Role-Differentiated Bimanual Movements Reflect Cognitive Impairments in Older Adults: A Cohort Sequential Study
Source: J Gerontol A Biol Sci Med Sci. 2024 Jun 24;79(10):glae137. doi: 10.1093/gerona/glae137 (PMC11372707; doi:10.1093/gerona/glae137)
Supplement: glae137_suppl_Supplementary_Materials [file glae137_suppl_supplementary_materials.docx]

S 1: Model fit for the linear mixed models for the depending variable Coefficient of Variation. Main and interaction effects were successively added to the model. The lowest AIC (bold) was used to determine the best mode fit.

| **Effect** | | **DF** | **AIC** | **logLik** | **Test** | **Likelihood ratio** | ***p* value** |
| --- | --- | --- | --- | --- | --- | --- | --- |
| Intercept | | 6 | 6288.1 | -3138.05 | - |  |  |
| Condition | | 7 | 6232.05 | -3109.03 | 1 vs 2 | 58.05 | <.001 |
| Group | | 8 | 6230.13 | -3107.06 | 2 vs 3 | 3.92 | .048 |
| Hand | | 9 | 6229.82 | -3105.91 | 3 vs 4 | 2.31 | .129 |
| Sex | | 10 | 6221.11 | -3100.56 | 4 vs 5 | 10.71 | .001 |
| Timepoint | | 11 | 6222.68 | -3100.34 | 5 vs 6 | 0.43 | .513 |
| Condition * Group | | 12 | 6224.31 | -3100.15 | 6 vs 7 | 0.37 | .541 |
| Condition * Hand | | 13 | **6213.28** | -3093.64 | 7 vs 8 | 13.03 | <.001 |
| Condition * Sex | | 14 | 6215.17 | -3093.59 | 8 vs 9 | 0.11 | .739 |
| Condition * Timepoint | | 15 | 6215.84 | -3092.92 | 9 vs 10 | 1.33 | .249 |
| Group * Hand | | 16 | 6217.46 | -3092.73 | 10 vs 11 | 0.39 | .535 |
| Group * Sex | | 17 | 6218.46 | -3092.23 | 11 vs 12 | 1.00 | .317 |
| Group * Timepoint | | 18 | 6219.23 | -3091.62 | 12 vs 13 | 1.22 | .269 |
| Hand * Sex | | 19 | 6217.4 | -3089.70 | 13 vs 14 | 3.83 | .050 |
| Hand * Timepoint | | 20 | 6215.36 | -3087.68 | 14 vs 15 | 4.04 | .044 |
| Sex * Timepoint | | 21 | 6217.21 | -3087.61 | 15 vs 16 | 0.15 | .698 |
| Condition * Group * Timepoint | | 22 | 6218.13 | -3087.06 | 16 vs 17 | 1.08 | .298 |
| Condition * Group * Hand | | 23 | 6218.04 | -3086.02 | 17 vs 18 | 2.09 | .148 |
|  | Note: DF = degrees of freedom; AIC = Akaike information criterion; LogLik = logarithm of the likelihood function; p value was determined from the likelihood ratios of two successive models | | | | | | |

S 2: Model fit for the linear mixed models for the depending variable DFA-α. Main and interaction effects were successively added to the model. The lowest AIC (bold) was used to determine the best mode fit.

| **Effect** | | **DF** | **AIC** | **logLik** | **Test** | **Likelihood ratio** | ***p* value** |
| --- | --- | --- | --- | --- | --- | --- | --- |
| Intercept | | 6 | -2012.93 | 1012.46 | - |  |  |
| Condition | | 7 | -2018.14 | 1016.07 | 1 vs 2 | 7.22 | .007 |
| Group | | 8 | -2016.22 | 1016.11 | 2 vs 3 | 0.07 | .789 |
| Hand | | 9 | -2018.33 | 1018.17 | 3 vs 4 | 4.12 | .042 |
| Sex | | 10 | -2046.72 | 1033.36 | 4 vs 5 | 30.39 | <.001 |
| Timepoint | | 11 | -2044.73 | 1033.36 | 5 vs 6 | 0.01 | .925 |
| Condition * Group | | 12 | -2045.33 | 1034.66 | 6 vs 7 | 2.60 | .107 |
| Condition * Hand | | 13 | -2045.49 | 1035.74 | 7 vs 8 | 2.16 | .142 |
| Condition * Sex | | 14 | -2058.03 | 1043.02 | 8 vs 9 | 14.55 | <.001 |
| Condition * Timepoint | | 15 | -2056.97 | 1043.48 | 9 vs 10 | 0.93 | .334 |
| Group * Hand | | 16 | **-2061.52** | 1046.76 | 10 vs 11 | 6.56 | .010 |
| Group * Sex | | 17 | -2060.61 | 1047.3 | 11 vs 12 | 1.08 | .299 |
| Group * Timepoint | | 18 | -2059.97 | 1047.98 | 12 vs 13 | 1.36 | .243 |
| Hand * Sex | | 19 | -2058.42 | 1048.21 | 13 vs 14 | 0.45 | .500 |
| Hand * Timepoint | | 20 | -2056.84 | 1048.42 | 14 vs 15 | 0.42 | .518 |
| Sex * Timepoint | | 21 | -2055.27 | 1048.64 | 15 vs 16 | 0.43 | .511 |
| Condition * Group * Timepoint | | 22 | -2053.31 | 1048.66 | 16 vs 17 | 0.04 | .835 |
| Condition * Group * Hand | | 23 | -2051.33 | 1048.67 | 17 vs 18 | 0.02 | .894 |
|  | Note: DF = degrees of freedom; AIC = Akaike information criterion; LogLik = logarithm of the likelihood function; p value was determined from the likelihood ratios of two successive models | | | | | | |

S 3: Model fit for the linear mixed models for the depending variable Sample Entropy. Main and interaction effects were successively added to the model. The lowest AIC (bold) was used to determine the best mode fit.

| **Effect** | | **DF** | **AIC** | **logLik** | **Test** | **Likelihood ratio** | ***p* value** |
| --- | --- | --- | --- | --- | --- | --- | --- |
| Intercept | | 6 | -1497.55 | 754.77 |  |  |  |
| Condition | | 7 | -1569.91 | 791.95 | 1 vs 2 | 74.36 | <.001 |
| Group | | 8 | -1568.68 | 792.34 | 2 vs 3 | 0.77 | .380 |
| Hand | | 9 | -1571.46 | 794.73 | 3 vs 4 | 4.78 | .029 |
| Sex | | 10 | -1571.38 | 795.69 | 4 vs 5 | 1.92 | .166 |
| Timepoint | | 11 | -1569.63 | 795.82 | 5 vs 6 | 0.25 | .618 |
| Condition*Group | | 12 | -1572.48 | 798.24 | 6 vs 7 | 4.85 | .028 |
| Condition*Hand | | 13 | **-1588.66** | 807.33 | 7 vs 8 | 18.18 | <.001 |
| Condition*Sex | | 14 | -1587.8 | 807.9 | 8 vs 9 | 1.14 | .286 |
| Condition*Timepoint | | 15 | -1585.91 | 807.95 | 9 vs 10 | 0.11 | .739 |
| Group*Hand | | 16 | -1585.86 | 808.93 | 10 vs 11 | 1.95 | .163 |
| Group*Sex | | 17 | -1584.15 | 809.07 | 11 vs 12 | 0.29 | .589 |
| Group*Timepoint | | 18 | -1582.27 | 809.14 | 12 vs 13 | 0.12 | .727 |
| Hand*Sex | | 19 | -1580.44 | 809.22 | 13 vs 14 | 0.17 | .680 |
| Hand*Timepoint | | 20 | -1578.46 | 809.23 | 14 vs 15 | 0.02 | .889 |
| Sex * Timepoint | | 21 | -1576.65 | 809.32 | 15 vs 16 | 0.19 | .667 |
| Condition*Group*Timepoint | | 22 | -1574.78 | 809.39 | 16 vs 17 | 0.13 | .715 |
| Condition*Group*Hand | | 23 | -1574.85 | 810.43 | 17 vs 18 | 2.07 | .150 |
|  | Note: DF = degrees of freedom; AIC = Akaike information criterion; LogLik = logarithm of the likelihood function; p value was determined from the likelihood ratios of two successive models | | | | | | |

S 4: Comparison of the model statistics for the dependent variable sample entropy when altering the window size m (2, 3 & 4). Results show that the results are robust and do not significantly change with different window sizes.

|  | **SEn: m = 2; r = 0.25** | | | | | **SEn: m = 3; r = 0.25** | | | | | **SEn: m = 4; r = 0.25** | | | | |
| --- | --- | --- | --- | --- | --- | --- | --- | --- | --- | --- | --- | --- | --- | --- | --- |
| **Predictors** | **Estimates** | **std.**  **Error** | **df** | **Statistic** | **p** | **Estimates** | **std.**  **Error** | **df** | **Statistic** | **p** | **Estimates** | **std.**  **Error** | **df** | **Statistic** | **p** |
| Intercept | 0.42 | 0.02 | 553 | 20.73 | **<.001** | 0.40 | 0.02 | 553 | 20.82 | **<.001** | 0.39 | 0.02 | 553 | 20.51 | **<.001** |
| Condition (RD-BC) | -0.11 | 0.01 | 129 | -7.50 | **<.001** | -0.10 | 0.01 | 129 | -7.69 | **<.001** | -0.10 | 0.01 | 129 | -7.66 | **<.001** |
| Group (MCI-CHI) | -0.04 | 0.02 | 128 | -1.91 | .058 | -0.04 | 0.02 | 128 | -1.96 | .053 | -0.04 | 0.02 | 128 | -1.97 | .050 |
| Hand (Right-Left) | 0.01 | 0.01 | 553 | 1.45 | .147 | 0.01 | 0.01 | 553 | 1.88 | .060 | 0.01 | 0.01 | 553 | 2.05 | **.040** |
| Sex (Female-Male) | -0.03 | 0.02 | 128 | -1.39 | .167 | -0.04 | 0.02 | 128 | -1.99 | **.048** | -0.04 | 0.02 | 128 | -1.90 | .060 |
| Timepoint | 0.00 | 0.01 | 293 | 0.50 | .620 | 0.00 | 0.00 | 293 | 0.71 | .476 | 0.00 | 0.00 | 293 | 0.68 | .500 |
| Condition (RD-BC)*Group (MCI-CHI) | 0.05 | 0.02 | 129 | 2.22 | **.028** | 0.05 | 0.02 | 129 | 2.32 | **.022** | 0.05 | 0.02 | 129 | 2.32 | **.022** |
| Condition (RD-BC)*Hand (Right-Left) | -0.04 | 0.01 | 553 | -4.28 | **<.001** | -0.04 | 0.01 | 553 | -4.43 | **<.001** | -0.04 | 0.01 | 553 | -4.47 | **<.001** |
| N | 2 _hand_ | | | | | 2 _hand_ | | | | | 2 _hand_ | | | | |
|  | 4 _tp_ | | | | | 4 _tp_ | | | | | 4 _tp_ | | | | |
|  | 2 _condition_ | | | | | 2 _condition_ | | | | | 2 _condition_ | | | | |
|  | 131 _id_ | | | | | 131 _id_ | | | | | 131 _id_ | | | | |
| Observations | 1111 | | | | | 1111 | | | | | 1111 | | | | |

S 5: Comparison of the model statistics for the dependent variable sample entropy when altering the threshold r (0.2, 0.25 & 0.3). Results show that the results are robust and do not significantly change with different thresholds.

|  | **SEn: m = 2; r = 0.2** | | | | | **SEn: m = 2; r = 0.25** | | | | | **SEn: m = 2; r = 0.3** | | | | |
| --- | --- | --- | --- | --- | --- | --- | --- | --- | --- | --- | --- | --- | --- | --- | --- |
| **Predictors** | **Estimates** | **std.**  **Error** | **df** | **Statistic** | **p** | **Estimates** | **std.**  **Error** | **df** | **Statistic** | **p** | **Estimates** | **std.**  **Error** | **df** | **Statistic** | **p** |
| Intercept | 0.50 | 0.02 | 553.00 | 21.19 | **<0.001** | 0.42 | 0.02 | 553.00 | 20.73 | **<0.001** | 0.34 | 0.02 | 553.00 | 20.07 | **<0.001** |
| Condition (RD-BC) | -0.13 | 0.02 | 129.00 | -7.80 | **<0.001** | -0.11 | 0.01 | 129.00 | -7.50 | **<0.001** | -0.09 | 0.01 | 129.00 | -7.29 | **<0.001** |
| Group (MCI-CHI) | -0.05 | 0.03 | 128.00 | -2.02 | **0.046** | -0.04 | 0.02 | 128.00 | -1.91 | 0.058 | -0.04 | 0.02 | 128.00 | -1.87 | 0.063 |
| Hand (Right-Left) | 0.02 | 0.01 | 553.00 | 1.97 | **0.049** | 0.01 | 0.01 | 553.00 | 1.45 | 0.147 | 0.01 | 0.01 | 553.00 | 1.46 | 0.144 |
| Sex (Female-Male) | -0.03 | 0.02 | 128.00 | -1.46 | 0.146 | -0.03 | 0.02 | 128.00 | -1.39 | 0.167 | -0.03 | 0.02 | 128.00 | -1.60 | 0.112 |
| Timepoint | 0.00 | 0.01 | 293.00 | 0.58 | 0.559 | 0.00 | 0.01 | 293.00 | 0.50 | 0.620 | 0.00 | 0.00 | 293.00 | 0.49 | 0.621 |
| Condition (RD-BC)*Group (MCI-CHI) | 0.06 | 0.02 | 129.00 | 2.33 | **0.021** | 0.05 | 0.02 | 129.00 | 2.22 | **0.028** | 0.04 | 0.02 | 129.00 | 2.18 | **0.031** |
| Condition (RD-BC)*Hand (Right-Left) | -0.05 | 0.01 | 553.00 | -4.47 | **<0.001** | -0.04 | 0.01 | 553.00 | -4.28 | **<0.001** | -0.04 | 0.01 | 553.00 | -4.23 | **<0.001** |
| N | 2 _hand_ | | | | | 2 _hand_ | | | | | 2 _hand_ | | | | |
|  | 4 _tp_ | | | | | 4 _tp_ | | | | | 4 _tp_ | | | | |
|  | 2 _condition_ | | | | | 2 _condition_ | | | | | 2 _condition_ | | | | |
|  | 131 _id_ | | | | | 131 _id_ | | | | | 131 _id_ | | | | |
| Observations | 1111 | | | | | 1111 | | | | | 1111 | | | | |

S 6: Comparison of model statistics for CV when excluding data from T3 & T4

|  | **CV (full dataset)** | | | | | **CV (excluding T3 & T4)** | | | | |
| --- | --- | --- | --- | --- | --- | --- | --- | --- | --- | --- |
| **Predictors** | **Estimates** | **std.**  **Error** | **df** | **Statistic** | **p** | **Estimates** | **std.**  **Error** | **df** | **Statistic** | **p** |
| Intercept | 4.33 | 0.67 | 553.00 | 6.44 | **<0.001** | 4.55 | 0.77 | 429.00 | 5.90 | **<0.001** |
| Condition (RD-BC) | 2.40 | 0.47 | 129.00 | 5.13 | **<0.001** | 2.24 | 0.53 | 129.00 | 4.21 | **<0.001** |
| Group (MCI-CHI) | 1.76 | 0.75 | 128.00 | 2.33 | **0.021** | 1.86 | 0.79 | 128.00 | 2.36 | **0.020** |
| Hand (Right-Left) | -0.36 | 0.24 | 553.00 | -1.47 | 0.142 | -0.51 | 0.29 | 429.00 | -1.75 | 0.081 |
| Sex (Female-Male) | 2.20 | 0.66 | 128.00 | 3.34 | **0.001** | 2.30 | 0.68 | 128.00 | 3.36 | **0.001** |
| Timepoint | -0.10 | 0.16 | 293.00 | -0.65 | 0.515 | -0.24 | 0.31 | 169.00 | -0.77 | 0.440 |
| Condition (RD-BC)*Group (MCI-CHI) | -0.42 | 0.69 | 129.00 | -0.61 | 0.540 | -0.50 | 0.76 | 129.00 | -0.65 | 0.515 |
| Condition (RD-BC)*Hand (Right-Left) | 1.24 | 0.34 | 553.00 | 3.62 | **<0.001** | 1.31 | 0.42 | 429.00 | 3.15 | **0.002** |
| N | 2 _hand_ | | | | | 2 _hand_ | | | | |
|  | 4 _tp_ | | | | | 2 _tp_ | | | | |
|  | 2 _condition_ | | | | | 2 _condition_ | | | | |
|  | 131 _id_ | | | | | 131 _id_ | | | | |
| Observations | 1111 | | | | | 863 | | | | |

S 7: Comparison of model statistics for DFA when excluding data from T3 & T4

|  | **DFA(full dataset)** | | | | | **DFA(excluding T3 & T4)** | | | | |
| --- | --- | --- | --- | --- | --- | --- | --- | --- | --- | --- |
| **Predictors** | **Estimates** | **std.**  **Error** | **df** | **Statistic** | **p** | **Estimates** | **std.**  **Error** | **df** | **Statistic** | **p** |
| Intercept | 1.30 | 0.02 | 552.00 | 66.10 | **<0.001** | 1.30 | 0.02 | 428.00 | 58.80 | **<0.001** |
| Condition (RD-BC) | 0.04 | 0.02 | 128.00 | 2.61 | **0.010** | 0.04 | 0.02 | 128.00 | 1.95 | 0.053 |
| Group (MCI-CHI) | 0.02 | 0.02 | 128.00 | 0.83 | 0.408 | 0.02 | 0.02 | 128.00 | 0.86 | 0.393 |
| Hand (Right-Left) | -0.01 | 0.01 | 552.00 | -0.89 | 0.376 | -0.01 | 0.01 | 428.00 | -0.67 | 0.503 |
| Sex (Female-Male) | 0.14 | 0.02 | 128.00 | 6.81 | **<0.001** | 0.14 | 0.02 | 128.00 | 6.51 | **<0.001** |
| Timepoint | -0.00 | 0.00 | 292.00 | -0.58 | 0.560 | 0.00 | 0.01 | 168.00 | 0.31 | 0.754 |
| Condition (RD-BC)*Group (MCI-CHI) | -0.03 | 0.01 | 128.00 | -2.07 | **0.040** | -0.03 | 0.01 | 128.00 | -2.06 | **0.041** |
| Condition (RD-BC)*Hand (Right-Left) | 0.01 | 0.01 | 552.00 | 1.47 | 0.141 | 0.01 | 0.01 | 428.00 | 1.34 | 0.180 |
| Condition (RD-BC)*Sex (Female-Male) | -0.05 | 0.01 | 128.00 | -4.04 | **<0.001** | -0.05 | 0.01 | 128.00 | -3.26 | **0.001** |
| Condition (RD)*Timepoint | 0.01 | 0.01 | 292.00 | 0.96 | 0.335 | 0.00 | 0.01 | 168.00 | 0.31 | 0.759 |
| Group (MCI-CHI)*Hand (Right-Left) | 0.02 | 0.01 | 552.00 | 2.56 | **0.011** | 0.03 | 0.01 | 428.00 | 2.55 | **0.011** |
| N | 2 _hand_ | | | | | 2 _hand_ | | | | |
|  | 4 _tp_ | | | | | 2 _tp_ | | | | |
|  | 2 _condition_ | | | | | 2 _condition_ | | | | |
|  | 131 _id_ | | | | | 131 _id_ | | | | |
| Observations | 1111 | | | | | 863 | | | | |

S 8: Comparison of model statistics for SEn when excluding data from T3 & T4

|  | **Sen (full dataset)** | | | | | **Sen (excluding T3 & T4)** | | | | |
| --- | --- | --- | --- | --- | --- | --- | --- | --- | --- | --- |
| **Predictors** | **Estimates** | **std.**  **Error** | **df** | **Statistic** | **p** | **Estimates** | **std.**  **Error** | **df** | **Statistic** | **p** |
| **Intercept** | 0.42 | 0.02 | 553.00 | 20.73 | **<0.001** | 0.42 | 0.02 | 429.00 | 17.87 | **<0.001** |
| **Condition (RD-BC)** | -0.11 | 0.01 | 129.00 | -7.50 | **<0.001** | -0.11 | 0.02 | 129.00 | -6.76 | **<0.001** |
| **Group (MCI-CHI)** | -0.04 | 0.02 | 128.00 | -1.91 | 0.058 | -0.05 | 0.02 | 128.00 | -2.07 | **0.040** |
| Hand (Right-Left) | 0.01 | 0.01 | 553.00 | 1.45 | 0.147 | 0.01 | 0.01 | 429.00 | 1.18 | 0.239 |
| **Sex (Female-Male)** | -0.03 | 0.02 | 128.00 | -1.39 | 0.167 | -0.03 | 0.02 | 128.00 | -1.55 | 0.123 |
| Timepoint | 0.00 | 0.01 | 293.00 | 0.50 | 0.620 | 0.01 | 0.01 | 169.00 | 0.50 | 0.621 |
| Condition (RD-BC)*Group (MCI-CHI) | 0.05 | 0.02 | 129.00 | 2.22 | **0.028** | 0.05 | 0.02 | 129.00 | 2.18 | **0.031** |
| **Condition (RD-BC)*Hand (Right-Left)** | -0.04 | 0.01 | 553.00 | -4.28 | **<0.001** | -0.04 | 0.01 | 429.00 | -3.78 | **<0.001** |
| N | 2 _hand_ | | | | | 2 _hand_ | | | | |
|  | 4 _tp_ | | | | | 2 _tp_ | | | | |
|  | 2 _condition_ | | | | | 2 _condition_ | | | | |
|  | 131 _id_ | | | | | 131 _id_ | | | | |
| Observations | 1111 | | | | | 863 | | | | |
